# The Association Between Intradialytic Symptom Clusters and Recovery Time in Patients Undergoing Maintenance Hemodialysis: An Exploratory Analysis

**DOI:** 10.1177/20543581241237322

**Published:** 2024-03-25

**Authors:** Arrti A. Bhasin, Jennifer M. MacRae, Braden Manns, Kelvin C. W. Leung, Amber O. Molnar, Jason W. Busse, David Collister, K Scott Brimble, Christian G. Rabbat, Jessica Tyrwhitt, Andrea Mazzetti, Michael Walsh

**Affiliations:** 1 Department of Health Research Methods, Evidence and Impact, McMaster University, Hamilton, ON, Canada; 2 Department of Medicine, University of Calgary, AB, Canada; 3 Department of Medicine, McMaster University, Hamilton, ON, Canada; 4 St. Joseph’s Healthcare Hamilton, ON, Canada; 5 Department of Anesthesia, McMaster University, Hamilton, ON, Canada; 6 Department of Medicine, University of Alberta, Edmonton, Canada; 7 Population Health Research Institute, Hamilton, ON, Canada

**Keywords:** hemodialysis, symptom clusters, recovery time, quality of life

## Abstract

**Background:**

Individuals receiving hemodialysis often experience concurrent symptoms during treatment and frequently report feeling unwell after dialysis. The degree to which intradialytic symptoms are related, and which specific symptoms may impair health-related quality of life (HRQoL) is uncertain.

**Objectives:**

To explore intradialytic symptoms clusters, and the relationship between intradialytic symptom clusters with dialysis treatment recovery time and HRQoL.

**Design/setting:**

We conducted a post hoc analysis of a prospective cohort study of 118 prevalent patients receiving hemodialysis in two centers in Calgary, Alberta and Hamilton, Ontario, Canada.

**Participants:**

Adults receiving hemodialysis treatment for at least 3 months, not scheduled for a modality change within 6 weeks of study commencement, who could provide informed consent and were able to complete English questionnaires independently or with assistance.

**Methods:**

Participants self-reported the presence (1 = *none* to 5 = *very much*) of 10 symptoms during each dialysis treatment, the time it took to recover from each treatment, and weekly Kidney Disease Quality of Life 36-Item–Short Form (KDQoL-36) assessments. Principal component analysis identified clusters of intradialytic symptoms. Mixed-effects, ordinal and linear regression examined the association between symptom clusters and recovery time (categorized as 0, >0 to 2, >2 to 6, or >6 hours), and the physical component and mental component scores (PCS and MCS) of the KDQoL-36.

**Results:**

One hundred sixteen participants completed 901 intradialytic symptom questionnaires. The most common symptom was lack of energy (56% of treatments). Two intradialytic symptom clusters explained 39% of the total variance of available symptom data. The first cluster included bone or joint pain, muscle cramps, muscle soreness, feeling nervous, and lack of energy. The second cluster included nausea/vomiting, diarrhea and chest pain, and headache. The first cluster (median score: −0.56, 25th to 75th percentile: −1.18 to 0.55) was independently associated with longer recovery time (odds ratio [OR] 1.62 per unit difference in score, 95% confidence interval [CI]: 1.23-2.12) and decreased PCS (−0.72 per unit difference in score, 95% CI: −1.29 to −0.15) and MCS scores (−0.82 per unit difference in score, 95% CI: −1.48 to −0.16), whereas the second cluster was not (OR 1.24, 95% CI: 0.97-1.58; PCS 0.19, 95% CI −0.46 to 0.83; MCS −0.72, 95% CI: −1.50 to 0.06).

**Limitations:**

This was an exploratory analysis of a small data set from 2 centers. Further work is needed to externally validate these findings to confirm intradialytic symptom clusters and the generalizability of our findings.

**Conclusions:**

Intradialytic symptoms are correlated. The presence of select intradialytic symptoms may prolong the time it takes for a patient to recover from a dialysis treatment and impair HRQoL.

## Abrégé

**Contexte:**

Il arrive fréquemment que les personnes qui reçoivent des traitements d’hémodialyse éprouvent des symptômes concomitants pendant la dialyze et signalent un malaise après le traitement. On en sait toutefois peu sur le degré de corrélation de ce malaise avec les symptômes intradialytiques et sur les symptômes précis qui peuvent altérer la qualité de vie liée à la santé (QVLS).

**Objectifs:**

Explorer différents groupes de symptômes intradialytiques et la relation de ceux-ci avec le temps de récupération post-dialyze et la QVLS.

**Cadre et conception de l’étude:**

Nous avons procédé à une analyze post-hoc d’une étude de cohorte prospective portant sur 118 patients prévalents recevant une hémodialyse dans deux centers, soit à Calgary (Alberta) et à Hamilton (Ontario) au Canada.

**Sujets:**

Des adultes qui recevaient des traitements d’hémodialyse depuis au moins trois mois — sans changement de modalité prévu dans les six semaines suivant le début de l’étude — qui pouvaient donner leur consentement éclairé et qui étaient en mesure de remplir des questionnaires en anglais de façon autonome ou avec de l’aide.

**Méthodologie:**

Pour chaque traitement de dialyze, les participants devaient autoévaluer le degré de présence (de 1 [non présent] à 5 [très présent]) de dix symptômes et le temps nécessaire pour récupérer de chaque traitement, puis remplir des évaluations hebdomadaires à l’aide du questionnaire KDQoL-36. Une analyze des composantes principales a permis de définir des groupes de symptômes intradialytiques. Une régression à effets mixtes, ordinale et linéaire, a servi à examiner l’association entre les groupes de symptômes et le temps de récupération (0 heure; de 0 à 2 heures; de 2 à 6 hures; plus de 6 heures), et les scores des composantes physiques et psychologiques du KDQoL-36.

**Résultats:**

Cent seize patients ont rempli un total de 901 questionnaires sur les symptômes intradialytiques. Le symptôme le plus fréquemment déclaré était le manque d’énergie (56 % des traitements). Deux groupes de symptômes intradialytiques ont expliqué 39 % de la variance totale des données disponibles sur les symptômes. Le premier groupe comprenait des douleurs osseuses ou articulaires, des crampes musculaires, des douleurs musculaires, une sensation de nervosité et un manque d’énergie. Le deuxième groupe comprenait des nausées/vomissements, de la diarrhée, des douleurs thoraciques et des maux de tête. Le premier groupe (score médian : –0,56; du 25^e^ au 75^e^ percentile : –1, 18 à 0,55) a été indépendamment associé à un temps de récupération plus long (rapport de cotes : 1,62 par unité de différence de score; IC 95 % : 1,23 à 2,12) et à une diminution des scores des composantes physiques (RC : –0,72; IC 95 % : –1, 29 à –0,15) et des scores des composantes psychologiques (RC : –0,82; IC 95 % : –1, 48 à –0,16). Le deuxième groupe n’a pas été associé avec le temps de récupération (RC : 1,24; IC 95 % : 0,97 à 1,58) ni avec le score des composantes physiques (RC : 0,19; IC 95 % : –0,46 à 0,83) et les scores des composantes psychologiques (RC : –0,72; IC 95 % : –1, 50 à 0,06).

**Limites:**

Il s’agissait d’une analyze exploratoire d’un petit ensemble de données provenant de deux centers. D’autres études externes sont nécessaires pour valider ces résultats et, ainsi, confirmer nos groupes de symptômes intradialytiques et la généralisabilité de nos résultats.

**Conclusion:**

Les symptômes intradialytiques sont corrélés. La présence de certains symptômes intradialytiques peut prolonger le temps de récupération post-dialyze et altérer la qualité de vie des patients.

## Introduction

Hemodialysis is a life-sustaining intervention; however, individuals often feel unwell during and after treatment. Hemodialysis-associated symptoms (eg, fatigue, cramping, body aches, and worry) are relatively common and can negatively impact an individual’s overall health-related quality of life (HRQoL).^1^ The management of dialysis-related symptoms has been identified as a research priority by both patients and physicians,^2^ and evaluating intradialytic symptoms, or symptoms occurring specifically during a dialysis treatment, is critical in shifting toward a patient-centered approach to dialysis delivery.^3^ Despite the recognized importance of symptom management and science in nephrology, major knowledge gaps exist regarding the assessment and patterns of symptom burden in chronic kidney disease.^4^

Individuals receiving dialysis treatment often report a multidimensional symptom burden where several symptoms occur concurrently and may share common causes.^5-9^ For example, events such as intradialytic hypotension (IDH) have been hypothesized to cause symptoms both during and after dialysis.^10^ Classifying symptoms into important groups or clusters of symptoms that are correlated or may have shared causes, may be more appropriate than measuring and managing symptoms separately.

While previous studies have explored symptom clusters in advanced kidney disease, they have largely focused on the overall symptom burden in this population, thus symptoms assessed may be rooted in causes other than dialysis treatment.^5-9,11-13^ Identifying symptom clusters that occur during dialysis treatment specifically is important as individuals with greater intradialytic symptom burden require more time to recover from feeling unwell after treatment.^14-16^ Consequentially, longer post-dialysis recovery time (>12 hours compared with 2-6 hours) is associated with hospitalization and mortality.^17^

To better understand the implications of dialysis-associated symptoms on HRQoL, we explored the degree to which intradialytic symptoms associate with one another in clusters, and how those intradialytic symptom clusters associate with recovery time immediately after a hemodialysis treatment.

## Methods

We conducted a post hoc, exploratory analysis of data from a prospective cohort study conducted in 2013. This original study was designed to understand the patient symptom experience and to evaluate different methods of measuring post-dialysis recovery time. Adults (18 years of age or older) receiving in-center hemodialysis for at least 90 days were followed over 3 months (Supplemental Figure S1). Individuals were not eligible if they were expected to change dialysis modality within 6 weeks of study entry or were unable to give consent or complete English-language questionnaires independently or with assistance. In the original study, each month for 1 week, participants were asked to report the degree to which they experienced intradialytic symptoms at the end of each hemodialysis treatment. Before commencing the subsequent hemodialysis treatment, participants were asked to report the time it took to recover from the previous treatment. The instrument used to measure recovery time in the original study randomly varied each month between (1) a single overall recovery time question, (2) 10 symptom-specific recovery time questions, and (3) 10 symptom-specific recovery times with the severity of each symptom. At the end of each week, participants completed the Kidney Disease Quality of Life 36-Item–Short Form (KDQoL-36) questionnaire. No alterations were made to hemodialysis prescriptions, or care. Participant demographics, comorbidities, and dialysis characteristics were collected at baseline. All participants provided informed consent. The study received approval at the research ethics board at each site and was in accordance with the Declaration of Helsinki. The reporting of this post hoc analysis follows the Strengthening the Reporting of Observational Studies in Epidemiology (STROBE) guidelines for observational studies (Supplemental Table S1).^18^

### Exposures and Outcomes

The original study administered an intradialytic symptom questionnaire that inquired about 10 symptoms at the end of each dialysis treatment: (1) nausea/vomiting, (2) diarrhea, (3) nervousness, (4) lack of energy, (5) muscle cramps, (6) shortness of breath, (7) muscle soreness, (8) bone or joint pain, (9) chest pain, and (10) headache. At the end of each dialysis treatment, participants were asked “Please think about how you felt today during your dialysis treatment. Did you have any of the following symptoms?” and were to report their experience with each symptom on a 5-point Likert scale (ie, *none, a little, somewhat, quite a bit*, or *very much*). This questionnaire was derived from the Dialysis Symptom Index^19^ by nephrologists and methodologists based on a pilot study measuring correlations with intradialytic systolic blood pressure and recovery time. For this post hoc analysis, IDH was defined as a nadir systolic blood pressure of <90 mmHg during the dialysis treatment.^20^

In the original study, a single overall recovery time question was found to be the most sensitive instrument.^21^ Therefore data from the weeks where this instrument was administered were used for the current analyses in which recovery time was the outcome. In this approach, participants were asked “How long did it take you to recover from your last dialysis session?” This was an adaptation of a previously proposed, open-ended recovery time measurement technique.^22^

Overall HRQoL was measured using the KDQoL-36 questionnaire, with a 7-day recall period. The questionnaire was administered to participants at the end of each index week. The generic component scores were of interest in this analysis, including the derived physical component score (PCS) and mental component score (MCS) of the Short Form-12 domains of the KDQoL-36. Possible scores ranged from 0 to 100, with higher scores indicating better HRQoL.^23,24^

### Statistical Analysis

Participant characteristics are presented as the mean and standard deviation (SD) if normally distributed, or median and interquartile range (IQR) if not, for continuous variables and as proportions for categorical data. Normality of continuous variables was assessed visually by normal probability plots, and statistically using the Shapiro-Wilk test. A complete case analysis of available data was conducted, where only data from fully completed symptom questionnaires and outcome measures were included in relevant analyses.

Principal component analysis (PCA) was used to reduce the complexity of our intradialytic symptom data from 10 individual symptoms to clusters, or groups, of correlated symptoms. The PCA reconstructs the data to create symptoms clusters based on linear combinations of correlated variables. Given the exploratory nature of our analyses, we did not make a priori assumptions of potential clusters of symptoms or the underlying latent cause(s) of clusters.

Bartlett’s test for sphericity and a Kaiser-Meyer-Olkin (KMO) value >0.6 were used to evaluate the adequacy of the intradialytic symptom data for PCA. Varimax rotation with Kaiser normalization was used to maximize the sum of the variances of squared loadings within each symptom cluster. Clusters were evaluated by visual inspection of a scree plot and their corresponding eigenvalues; only clusters with eigenvalues >1, or symptom clusters contributing to more of the variance of the data than individual symptoms, were selected for further analyses. Individual symptoms were retained in each cluster if they had a factor loading of >0.3 (i.e., at least a *moderate* correlation with the cluster itself). Cronbach’s alpha was used to further assess the internal consistency of each identified symptom cluster by PCA.

We constructed mixed-effects multivariable ordinal logistic regression models to assess the relationship between intradialytic symptom clusters and recovery time, in which recovery time was the dependent variable and participants were considered random intercepts to account for repeated observations. We categorized recovery time as 0, >0 to 2, >2 to 6, >6 hours on the basis of its distribution as a continuous outcome, and previous work.^17,21^ Each symptom cluster score was treated as a continuous variable based on the weighted loading calculated from every reported symptom for each participant’s dialysis treatment from PCA.

We constructed mixed-effects multivariable linear regression models to evaluate the association between symptom cluster scores (independent variables) and the PCS and MCS subscales of the KDQoL-36 (dependent variables), with participants treated as a random intercept. Linear regression models were evaluated by visual inspection of residual plots. Model goodness of fit was summarized by corresponding Akaike information criterion (AIC) values, with lower AIC values representing a better fit.

All models were adjusted for participant factors that may be associated with symptom experience, recovery time, HRQoL, and general dialysis practices. These variables were selected a priori, based on clinical expertise and prior evidence,^17^ and included participant age in years (continuous), sex (female vs male), dialysis center (Hamilton vs Calgary), vintage (years receiving dialysis, continuous), comorbidities including history of stroke, coronary artery disease (CAD) or peripheral vascular disease (PVD), diabetes, and IDH. As IDH may cause symptoms both during and after dialysis,^10^ we used regression analysis to assess the relationship between IDH and intradialytic symptoms clusters with regard to recovery time. This was done by constructing models adjusted for IDH and symptom cluster scores individually and simultaneously.^25^ Sensitivity analysis was conducted, excluding individuals scheduled to receive more than 3 dialysis treatments per week.

Associations are presented as point estimates, with corresponding 95% confidence intervals (CIs). *P* values less than .05 were considered statistically significant. All analyses were performed using Stata version 14 (College Station, Texas).

## Results

### Participants

**Figure 1.**
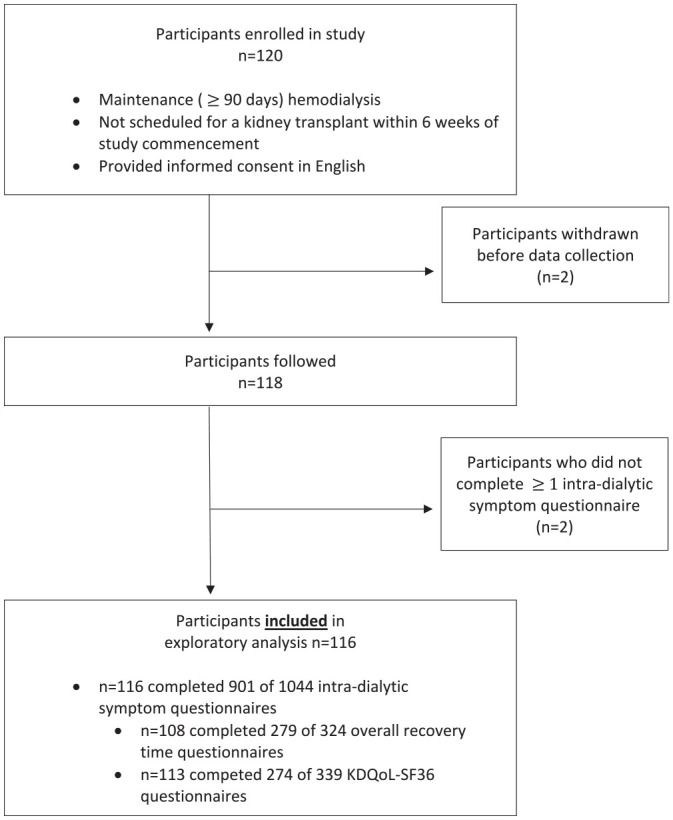
Participant flowchart.

Of the 120 participants who were enrolled, 2 withdrew consent prior to study commencement and 2 did not complete any intradialytic questionnaires, allowing 116 (97%) participants to be included in this analysis (Figure 1). Of the 116 participants with completed intradialytic symptom questionnaire(s), 108 participants also provided corresponding dialysis treatment recovery time data. Baseline demographic and clinical characteristics are summarized in Table 1. The median age was 68 years (IQR 58-77), and participants had a median dialysis vintage of 4 years (IQR 2-6). In the entire cohort, 38% (n = 44) of participants were female, and 76% (n = 88) were of Caucasian descent. Seventy-six percent of participants (n = 88) were scheduled to dialyze 3 times a week for a median of 4 hours (IQR 3.5-4). Of the 116 participants, one patient died during the study follow-up period, but completed at least one intradialytic symptom, recovery time, and the KDQoL-36 questionnaire.

**Table 1.** Baseline Cohort Characteristics.

Characteristics	Entire cohort (N = 116)	Maximum reported recovery time (N = 108)			
		0 hours (N = 9)	>0 to 2 hours (N = 33)	>2 to 6 hours (N = 27)	>6 hours (N = 39)
Age (y)	68 (58-77)	79 (68-81)	65 (54-72)	68 (62.5-76)	69 (60.5-78)
Female	44 (37.9%)	3 (33.3%)	11 (33.3%)	12 (44.4%)	16 (41.0%)
Dialysis vintage (y)	4 (2-6)	3 (2-3)	4 (1-4)	4 (2.5-8.5)	3 (2-5.5)
Ethnicity					
Caucasian	88 (75.9%)	8 (88.9%)	25 (75.8%)	19 (70.4%)	31 (79.5%)
Non-Caucasian	28 (24.1%)	1 (11.1%)	8 (24.2%)	8 (29.6%)	8 (20.5%)
Cause of kidney failure					
Diabetic Nephropathy	51 (44.0%)	5 (55.6%)	15 (45.5%)	7 (25.9%)	21 (53.9%)
Hypertension	15 (12.9%)	2 (22.2%)	3 (9.1%)	5 (18.5%)	4 (10.3%)
Glomerulonephritis	26 (22.4%)	2 (22.2%)	7 (21.2%)	7 (25.9%)	8 (20.5%)
Polycystic kidney disease	3 (2.6%)	0 (0.0%)	2 (6.1%)	1 (3.7%)	0 (0.0%)
Other	21 (18.1%)	0 (0.0%)	6 (18.2%)	7 (25.9%)	6 (15.4%)
History of:					
CAD or PVD	69 (59.5%)	7 (77.8%)	18 (54.5%)	10 (37.0%)	29 (74.4%)
CAD	51 (44.0%)	3 (33.3%)	13 (39.4%)	8 (29.6%)	19 (48.7%)
PVD	42 (36.2%)	6 (66.7%)	10 (30.3%)	8 (29.6%)	15 (38.5%)
Stroke	13 (11.2%)	1 (11.1%)	6 (18.2%)	2 (7.4%)	3 (7.7%)
Diabetes	60 (51.7%)	4 (44.4%)	20 (60.6%)	10 (37.0%)	23 (59.0%)
Dialysis prescription at baseline:					
Number of scheduled HD treatments					
Two/wk	2 (1.7%)	0 (0.0%)	0 (0.0%)	0 (0.0%)	0 (0.0%)
Three/wk	88 (75.9%)	9 (100.0%)	21 (63.6%)	23 (85.2%)	32 (82.1%)
Four/wk	10 (8.6%)	0 (0.0%)	5 (15.2%)	1 (3.7%)	4 (10.3%)
Five/wk	5 (4.3%)	0 (0.0%)	4 (12.1%)	1 (3.7%)	0 (0.0%)
Six/wk	11 (9.5%)	0 (0.0%)	3 (9.1%)	2 (7.4%)	3 (7.7%)
Length of treatment (hours)	4 (3.5-4)	4 (3.5-4)	4 (3.5-4)	4 (3.5-4)	3.5 (3.5-4)
Dialysate composition					
Sodium (mmol/L)	142 (140-142)	142 (140-142)	140 (138-142)	142 (141-142)	142 (138-142)
Calcium (mmol/L)	1.3 (1.3-1.3)	1.3 (1.3-1.3)	1.3 (1.3-1.3)	1.3 (1.3-1.3)	1.3 (1.3-1.3)
Potassium (mmol/L)	2.3 (2-3)	2.3 (2-3)	2.3 (2-3)	2.3 (2-3)	2.3 (2-3)
Glucose (mmol/L)	11.1 (11.1-11.1)	11.1 (8.3-11.1)	11.1 (8.3-11.1)	11.1 (11.1-11.1)	11.1 (11.1-11.1)
Sodium profile	11 (9.5%)	0 (0%)	3 (9.1%)	2 (7.4%)	6 (15.4%)
Ultrafiltration profile	18 (15.5%)	1 (11.1%)	4 (12.1%)	5 (18.5%)	8 (20.5%)
Dialysate temperature (°C)	36.5 (36.1-36.5)	36.5 (36.1-36.5)	36.3 (36-36.5)	36.5 (36.5-36.5)	36.5 (36.5-36.5)
Center					
Hamilton	77 (66.4%)	6 (66.7%)	18 (54.6%)	20 (74.1%)	27 (69.2%)
Calgary	39 (33.6%)	3 (33.3%)	15 (45.5%)	7 (25.9%)	12 (30.8%)

*Note*. CAD = coronary artery disease; HD = hemodialysis; PVD = peripheral vascular disease.

### Intradialytic Symptoms Clusters

**Figure 2.**
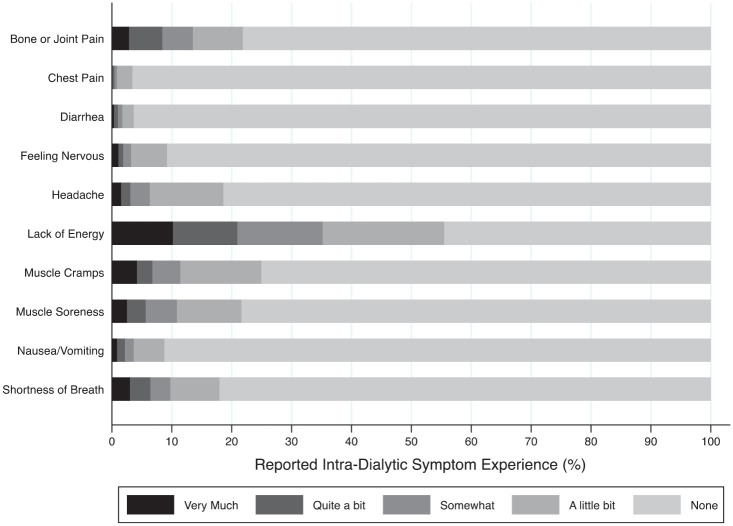
Distribution of intradialytic symptoms over 901 treatments in 116 participants.

A total of 901 intradialytic symptom questionnaires were completed (median 9 questionnaires per participant, IQR 7-9). The intradialytic symptom most reported was lack of energy, occurring during 56% of the dialysis treatments. This was followed by muscle cramps (25%), bone or joint pain (22%), and muscle soreness (22%). Lack of energy (10%), muscle cramps (4%), shortness of breath (3%), bone or joint pain (3%), and muscle soreness (3%) were the symptoms most often reported as “very much” present. Chest pain and diarrhea were the intradialytic symptoms least often experienced, reported during only 3% and 4% of all treatments (Figure 2, Supplemental Table S2).

**Figure 3.**
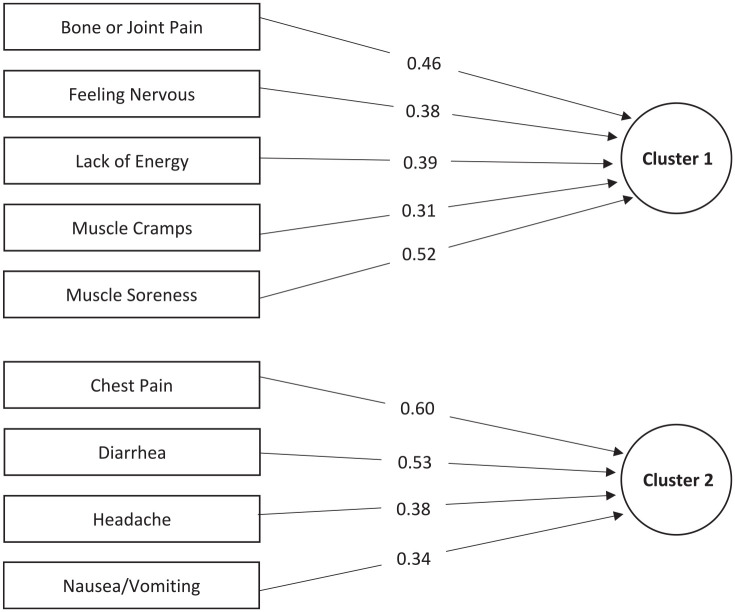
Intradialytic symptom clusters. Includes symptoms with factor loadings >0.3.

The data were adequate for PCA (KMO = 0.78, Bartlett’s test of sphericity *P* value < .001). The 10 individual intradialytic symptoms were reduced to two clusters of symptoms, explaining 39% of the total variance of the symptom data (Supplemental Table S3). Shortness of breath was the only symptom that did not significantly load onto a cluster (factor loading <0.3). The primary intradialytic symptom cluster, or symptom cluster explaining the greatest amount of variance, included bone or joint pain, feeling nervous, lack of energy, muscle cramps, and muscle soreness. The second cluster consisted of chest pain, diarrhea, headache, and nausea/vomiting (Figure 3). Clusters 1 and 2 had moderate and low internal consistency (Cronbach’s α = .64 and .40; Supplemental Table S3).

### Relationship Between Intradialytic Symptoms and Recovery Time

Recovery time was reported by 108 participants (Table 2) across 279 dialysis treatments and ranged from 0 to 44 hours. Forty-one (38%) participants reported a median recovery time between >0 and 2 hours across all dialysis treatments, and 39 (36%) participants reported at least one recovery time lasting >6 hours. Twenty-nine (27%) participants experienced IDH over 42 treatments (15% of all treatments).

**Table 2.** Distribution of Reported Outcomes.

Outcome	Entire cohort
Recovery Time	N = 108
Median recovery time (hours)	2 (0.1-5.0)
0 hours	18 (16.7%)
>0-2 hours	41 (38.0%)
>2-6 hours	25 (23.2%)
>6 hours	24 (22.2%)
Maximum recovery time (hours)	3 (1.0-24.0)
0 hours	9 (8.3%)
>0-2 hours	33 (30.6%)
>2-6 hours	27 (25.0%)
>6 hours	39 (36.1%)
HRQoL (KDQoL-36)	N = 113
Median PCS score	39.9 (35.8-43.7)
Median MCS score	45.7 (41.2-49.9)

*Note*. Presented as median (interquartile range) or n (%). HRQoL = health-related quality of life; KDQoL-36 = Kidney Disease Quality of Life 36-Item–Short Form; MCS = mental component score; PCS = physical component score.

The median scores were −0.56 (IQR –1.18 to 0.55) for symptom cluster 1, and −0.45 (IQR −0.61 to 0.08) for symptom cluster 2. More severe symptoms that comprise cluster 1 were associated with longer recovery time (odds ratio [OR] 1.62 per unit difference in score, 95% CI: 1.23-2.12) (Table 3). The association between more severe symptoms in cluster 2 and recovery time was not significant (OR 1.24 per unit difference in score, 95% CI: 0.97-1.58). Recovery time models improved with addition of intradialytic symptom clusters (AIC 708 vs 693; Supplemental Table S4). Direction and magnitude of associations between intradialytic symptom clusters and recovery time were similar when individuals who were scheduled to receive more frequent dialysis sessions (>3 sessions per week) were excluded (cluster 1: OR 2.11, 95% CI: 1.48-3.01; cluster 2: OR 1.00, 95% CI: 0.72-1.38).

**Table 3.** Associations Between HRQoL Outcomes and Symptom Clusters After Adjustment of Intradialytic Hypotension and Participant Characteristics Using Mixed-Effects Models.

Variables	Recovery time OR (95% CI)	PCS B (95% CI)	MCS B (95% CI)
Symptom cluster component 1	1.62 (1.23-2.12)	−0.72 (−1.29 to −0.15)	−0.82 (−1.48 to −0.16)
Symptom cluster component 2	1.24 (0.97-1.58)	0.19 (−0.46 to 0.83)	−0.72 (−1.50 to 0.06)
Intradialytic hypotension (sys <90 mm Hg)	2.13 (0.84-5.43)	0.26 (−2.27 to 2.79)	1.19 (−1.85 to 4.23)

*Note*. All models are adjusted for participant age (years, continuous), sex (female vs male), center (Calgary vs Hamilton), dialysis vintage (years, continuous), history of CAD (coronary artery disease) or PVD (peripheral vascular disease), history of stroke, and history of diabetes. B = beta coefficient from linear regression models; CI = confidence interval; HRQoL = health-related quality of life; MCS = mental component score; OR = odds ratio from mixed-effects ordinal regression model with recovery time categorized as an ordinal variable; PCS = physical component score.

### Relationship Between Intradialytic Symptoms and HRQoL

One hundred thirteen participants completed 274 KDQoL-36 questionnaires (Table 2). Symptom cluster 1 was associated with lower PCS (−0.72 per unit difference in symptom score, 95% CI: −1.29 to −0.15) and lower MCS scores (−0.82 per unit difference in symptom score, 95% CI: −1.48 to −0.16; Table 3). Symptom cluster 2 was not significantly associated with either PCS or MCS. The MCS models were more parsimonious when symptom clusters were included but the PCS models were not materially improved (Supplemental Tables S5 and S6).

### Relationship Between Intradialytic Symptom Clusters, IDH, and Recovery Time

IDH was associated with symptom cluster 2 (0.53 increase in symptom score in the presence of IDH compared with its absence, 95% CI: 0.04-1.03) and longer recovery time (OR 3.01 in the presence of IDH compared with its absence, 95% CI: 1.18-7.67; Supplemental Table S7). When both symptom cluster scores and IDH were included as covariates, IDH was no longer significantly associated with longer recovery time, but the magnitude of the association was only marginally attenuated (OR 2.47, 95% CI: 0.96-6.34). Intradialytic hypotension was not associated with symptom cluster 1, therefore recovery time models with and without IDH and symptom cluster 1 were not explored (Supplemental Table S8).

## Discussion

Our study found that individuals undergoing maintenance hemodialysis commonly report concurrent symptoms during treatment that may be grouped into 2 symptom clusters. The presence of certain intradialytic symptoms, including bone or joint pain, feeling nervous, lack of energy, muscle cramps, and muscle soreness, may increase the likelihood of prolonged recovery time and impair HRQoL more so than other symptoms. Furthermore, clustering of intradialytic symptoms may indicate shared pathophysiology, although further research is required to better delineate any shared causes.

Several studies have noted that individuals feel unwell during and after dialysis. A study of 623 prevalent adults receiving hemodialysis in the United Kingdom described tiredness, feeling cold, and muscle cramps commonly during dialysis and, whereas half of the patients recovered within 1 hour of completing dialysis, 20% required more than 4 hours.^15^ Rayner and colleagues reported that 27% of patients experienced a recovery time of more than 6 hours.^17^ Others have also reported that patients with longer post-dialysis recovery times have more impaired HRQoL.^14,15,26^

A recent cross-sectional survey in the United States found fatigue or feeling washed out or drained, cramps, and symptoms of low blood pressure were symptoms most commonly reported by individuals during hemodialysis treatment; vomiting and chest pain were least commonly reported.^16^ Of the entire cohort, 40% reported 4 or more hours of post-dialysis recovery time, and a higher number of symptoms and greater total symptom score (calculated by multiplying the number of symptoms by the average of the severity of symptoms) were correlated with longer recovery time.^16^ Our findings extend on previous work by identifying specific groups of intradialytic symptoms that are associated with prolonged recovery time.

Our observation of symptom clustering supports the concept that hemodialysis induces systemic events with widespread effects. Dialysis-induced hypotension is commonly considered a trigger of ischemic insults in the heart, brain, or gut, and is therefore rational to consider as a cause of nausea, vomiting, diarrhea, chest pain, and headaches with IDH.^27,28^ This may explain the clustering of such symptoms in cluster 2 and the association between IDH and this cluster. Importantly, as the risk of IDH is modifiable, the risk of some symptoms may also be modifiable.^29,30^ However, we found that the degree to which the association of IDH on recovery time is affected by intradialytic symptoms may, at best, be small. This suggests that the causal pathway between intradialytic symptoms and recovery time is unlikely to be rooted in IDH (within the limitations of our definition of IDH). Other pathophysiology should be explored with the caveats that any true associations may be obscured by the relatively crude definition of IDH and the inability to assess the timing of IDH relative to onset of symptoms. This finding suggests that alleviating intradialytic symptoms may require innovative strategies, such as intradialytic exercise and cognitive behavioral therapy, which are effective for symptom management in other settings.^31,32^

### Strengths and Limitations

Our study has several strengths. Although symptom clusters in people receiving hemodialysis have been previously reported,^5-9,11,12^ few have described clustering of intradialytic symptoms, which increases our understanding of potential shared causes and power to detect associations with outcomes. Previous studies of the relationship of intradialytic symptoms and recovery time were limited by reverse causation and recall biases due to their cross-sectional survey designs.^14-16^ Our findings are less susceptible to these issues by nature of the prospective assessments with short, well-defined recall periods.

The limitations of our study must also be acknowledged. First, as PCA does not allow for the quantitative interpretation of symptom cluster scores, the results are limited to qualitative interpretations of regression analysis. It also does not directly inform the underlying cause of each symptom cluster. These limitations do not negate the potential utility of identifying symptom clusters but do highlight the need for replication and extension of this work using confirmatory analyses and alternative symptom assessment tools. In addition, although the sample data were adequate for PCA, the symptom component scores may lack variability and the sample is small to precisely estimate an association between IDH and intradialytic symptoms. Finally, because so little is known about the causes of intradialytic symptoms and recovery time, and because our study is relatively small and other symptoms may be present, residual confounding and imprecision may substantially influence the results. Further work is needed to externally validate our findings and understand the underlying causes of these symptoms and their clustering.

## Conclusion

Intradialytic symptoms are correlated and may share a common cause. Bone or joint pain, feeling nervous, lack of energy, muscle cramps, and muscle soreness appear closely related and may be more important in determining recovery time and HRQoL than other intradialytic symptoms we measured. Further research is needed to confirm and identify the latent cause(s) of correlated intradialytic symptoms, and to evaluate the effect of innovative symptom management strategies on intradialytic symptom clusters and post-dialysis recovery time.

## Supplemental Material

sj-docx-1-cjk-10.1177_20543581241237322 – Supplemental material for The Association Between Intradialytic Symptom Clusters and Recovery Time in Patients Undergoing Maintenance Hemodialysis: An Exploratory Analysis Supplemental material, sj-docx-1-cjk-10.1177_20543581241237322 for The Association Between Intradialytic Symptom Clusters and Recovery Time in Patients Undergoing Maintenance Hemodialysis: An Exploratory Analysis by Arrti A. Bhasin, Jennifer M. MacRae, Braden Manns, Kelvin C. W. Leung, Amber O. Molnar, Jason W. Busse, David Collister, K Scott Brimble, Christian G. Rabbat, Jessica Tyrwhitt, Andrea Mazzetti and Michael Walsh in Canadian Journal of Kidney Health and Disease
