# Supplementary material for: The Association Between Intradialytic Symptom Clusters and Recovery Time in Patients Undergoing Maintenance Hemodialysis: An Exploratory Analysis
Source: Can J Kidney Health Dis. 2024 Mar 25;11:20543581241237322. doi: 10.1177/20543581241237322 (PMC10964465; doi:10.1177/20543581241237322)
Supplement: sj-docx-1-cjk-10.1177_20543581241237322 – Supplemental material for The Association Between Intradialytic Symptom Clusters and Recovery Time in Patients Undergoing Maintenance Hemodialysis: An Exploratory Analysis [file sj-docx-1-cjk-10.1177_20543581241237322.docx]

**Supplemental Material**

Supplemental Table 1 STROBE Reporting Checklist

Detailed classifications of select variables

Supplemental Table 2 Distribution of Reported Intra-dialytic Symptom Experience

Supplemental Table 3 Principal Component Analysis Factor Loadings/Scoring Coefficients

Supplemental Table 4 Recovery Time Mixed Ordinal Regression Models

Supplemental Table 5 PCS Mixed Linear Regression Models

Supplemental Table 6 MCS Mixed Linear Regression Models

Supplemental Table 7 Relationship between Intra-dialytic Symptom Clusters, Intra-dialytic Hypotension and Recovery Time

Supplemental Table 8 Symptom Cluster Component Scores and Intra-dialytic Hypotension from Mixed-effects Linear Regression Analysis

Supplemental Table 9 Recovery Time Models with Individual Symptoms

Supplemental Figure 1: Overview of Original Study Schedule

Supplemental Figure 2: Principal Component Analysis Scree Plot

Detailed classifications of select variables:

Coronary artery disease (CAD)

Defined as a history of one or more of the following conditions: myocardial infarction, unstable angina, coronary artery bypass graft surgery, percutaneous transluminal coronary angioplasty or atherectomy or percutaneous coronary intervention, heart failure, and/or carotid endarterectomy or stenting

Peripheral vascular disease (PVD)

Defined as history of intermittent claudication, foot infection or foot ulcer requiring antibiotic, limb or foot amputation, and peripheral artery surgery or angioplasty

Intra-dialytic Hypotension (IDH)

Defined as nadir systolic blood pressure of <90 mmHg. This definition is reportedly most associated with mortality.^1^ Across a given index week, intra-dialytic hypotension was defined as the presence of nadir systolic blood pressure of <90 mmHg in ≥ 50% of dialysis treatments in that week.

Recovery time

Participants were requested to indicate the unit of time of their recovery time response (minutes, hours, or days). Participant responses given in minutes were divided by 60 and responses given in days were divided by 24 respectively, to ensure all units of recovery time analyzed were in hours. The conventional time between one dialysis treatment to the next was 44 hours. Thus, all reported recovery times greater than this interval were replaced with a value of 44. In the instance where a participant indicated a unit of time (hours, minutes or days) but failed to provide an overall numerical estimate, recovery time was imputed with a value of 0.

^1^ Flythe JE, Xue H, Lynch KE, Curhan GC, Brunelli SM: Association of mortality risk with various definitions of intradialytic hypotension. *J Am Soc Nephrol,* 26**:** 724-734, 2015

Supplemental Table 1 STROBE Checklist^2^

STROBE Statement—Checklist of items that should be included in observational studies

|  | Item No. | Recommendation | Page  No. |
| --- | --- | --- | --- |
| **Title and abstract** | 1 | (*a*) Indicate the study’s design with a commonly used term in the title or the abstract | 1,3 |
|  |  | (*b*) Provide in the abstract an informative and balanced summary of what was done and what was found | 3,4 |
| Introduction | | | |
| Background/rationale | 2 | Explain the scientific background and rationale for the investigation being reported | 5,6 |
| Objectives | 3 | State specific objectives, including any prespecified hypotheses | 6 |
| Methods | | | |
| Study design | 4 | Present key elements of study design early in the paper | 6,7,  Supp Fig 1 |
| Setting | 5 | Describe the setting, locations, and relevant dates, including periods of recruitment, exposure, follow-up, and data collection | 6,7,  Supp Fig 1 |
| Participants | 6 | (*a*) *Cohort study*—Give the eligibility criteria, and the sources and methods of selection of participants. Describe methods of follow-up  *Case-control study*—Give the eligibility criteria, and the sources and methods of case ascertainment and control selection. Give the rationale for the choice of cases and controls  *Cross-sectional study*—Give the eligibility criteria, and the sources and methods of selection of participants | 6,7,  Supp Fig 1 |
|  |  | (*b*) *Cohort study*—For matched studies, give matching criteria and number of exposed and unexposed  *Case-control study*—For matched studies, give matching criteria and the number of controls per case | 6,7,  Supp Fig 1 |
| Variables | 7 | Clearly define all outcomes, exposures, predictors, potential confounders, and effect modifiers. Give diagnostic criteria, if applicable | 6,7,  Supp Fig 1 |
| Data sources/ measurement | 8* | For each variable of interest, give sources of data and details of methods of assessment (measurement). Describe comparability of assessment methods if there is more than one group | 6-8, 10 |
| Bias | 9 | Describe any efforts to address potential sources of bias | 6-8 |
| Study size | 10 | Explain how the study size was arrived at | 6-8, Supp Fig 2 |
| Quantitative variables | 11 | Explain how quantitative variables were handled in the analyses. If applicable, describe which groupings were chosen and why | 6-10 |
| Statistical methods | 12 | (*a*) Describe all statistical methods, including those used to control for confounding | 8-10 |
|  |  | (*b*) Describe any methods used to examine subgroups and interactions | NA |
|  |  | (*c*) Explain how missing data were addressed | 8, Supp |
|  |  | (*d*) *Cohort study*—If applicable, explain how loss to follow-up was addressed  *Case-control study*—If applicable, explain how matching of cases and controls was addressed  *Cross-sectional study*—If applicable, describe analytical methods taking account of sampling strategy | 8, Supp |
|  |  | (*e*) Describe any sensitivity analyses | NA |
| **Results** | | | |
| Participants | 13* | (a) Report numbers of individuals at each stage of study—eg numbers potentially eligible, examined for eligibility, confirmed eligible, included in the study, completing follow-up, and analysed | 10-12, Supp Fig 2 |
|  |  | (b) Give reasons for non-participation at each stage | NA |
|  |  | (c) Consider use of a flow diagram | Supp Fig 2 |
| Descriptive data | 14* | (a) Give characteristics of study participants (eg demographic, clinical, social) and information on exposures and potential confounders | 10, 11, Table 1 |
|  |  | (b) Indicate number of participants with missing data for each variable of interest | Supp Fig 2 |
|  |  | (c) *Cohort study*—Summarise follow-up time (eg, average and total amount) | 8,  Supp Fig 1 |
| Outcome data | 15* | *Cohort study*—Report numbers of outcome events or summary measures over time | 11-12 Table 2, Supp Table 2 |
|  |  | *Case-control study—*Report numbers in each exposure category, or summary measures of exposure |  |
|  |  | *Cross-sectional study—*Report numbers of outcome events or summary measures |  |
| Main results | 16 | (*a*) Give unadjusted estimates and, if applicable, confounder-adjusted estimates and their precision (eg, 95% confidence interval). Make clear which confounders were adjusted for and why they were included | Table 3, Supp Tables 4, 6-9 |
|  |  | (*b*) Report category boundaries when continuous variables were categorized | 9 |
|  |  | (*c*) If relevant, consider translating estimates of relative risk into absolute risk for a meaningful time period | NA |
| Other analyses | 17 | Report other analyses done—eg analyses of subgroups and interactions, and sensitivity analyses | 13,  Supp Tables 5,6 |
| **Discussion** | | | |
| Key results | 18 | Summarise key results with reference to study objectives | 13 |
| Limitations | 19 | Discuss limitations of the study, taking into account sources of potential bias or imprecision. Discuss both direction and magnitude of any potential bias | 15 |
| Interpretation | 20 | Give a cautious overall interpretation of results considering objectives, limitations, multiplicity of analyses, results from similar studies, and other relevant evidence | 16 |
| Generalisability | 21 | Discuss the generalisability (external validity) of the study results | 15 |
| **Other information** | | | |
| Funding | 22 | Give the source of funding and the role of the funders for the present study and, if applicable, for the original study on which the present article is based | NA |

^2^von Elm E, Altman DG, Egger M, et al. The Strengthening the Reporting of Observational Studies in Epidemiology (STROBE) statement: guidelines for reporting observational studies. *J Clin Epidemiol*. 2008;61(4):344-349.

Supplemental Table 2 Distribution of Reported Intra-dialytic Symptom Experience

| **Symptom** | **None** | **A little** | **Somewhat** | **Quite a bit** | **Very Much** |
| --- | --- | --- | --- | --- | --- |
| Bone or Joint Pain | 704 (78.1%) | 75 (8.3%) | 46 (5.1%) | 50 (5.6%) | 26 (2.9%) |
| Chest Pain | 870 (96.6%) | 23 (2.6%) | 4 (0.4%) | 4 (0.4%) | 0 (0.0%) |
| Diarrhea | 868 (96.3%) | 17 (1.9%) | 7 (0.8%) | 6 (0.7%) | 3 (0.3%) |
| Feeling Nervous | 818 (90.8%) | 54 (6.0%) | 12 (1.3%) | 7 (0.8%) | 10 (1.1%) |
| Headache | 733 (81.4%) | 111 (12.3%) | 29 (3.2%) | 14 (1.6%) | 14 (1.6%) |
| Lack of Energy | 401 (44.5%) | 183 (20.3%) | 128 (14.2%) | 97 (10.8%) | 92 (10.2%) |
| Muscle Cramps | 676 (75.0%) | 122 (13.5%) | 42 (4.7%) | 23 (2.6%) | 38 (4.2%) |
| Muscle Soreness | 706 (78.4%) | 97 (10.8%) | 47 (5.2%) | 28 (3.1%) | 23 (2.6%) |
| Nausea/Vomiting | 822 (91.2%) | 46 (5.1%) | 13 (1.4%) | 12 (1.3%) | 8 (0.9%) |
| Shortness of Breath | 739 (82.0%) | 74 (8.2%) | 30 (3.3%) | 31 (3.4%) | 27 (3.0%) |

Supplemental Table 3 Principal Component Analysis Factor Loadings/Scoring Coefficients

| **Symptom** | **Cluster Component 1** | **Cluster Component 2** |
| --- | --- | --- |
| Bone or Joint Pain | 0.46 | -0.07 |
| Chest Pain | -0.14 | 0.60 |
| Diarrhea | -0.10 | 0.53 |
| Feeling Nervous | 0.38 | -0.01 |
| Headache | 0.12 | 0.38 |
| Lack of Energy | 0.39 | 0.11 |
| Muscle Cramps | 0.31 | 0.18 |
| Muscle Soreness | 0.52 | -0.11 |
| Nausea/Vomiting | 0.11 | 0.34 |
| Shortness of Breath | 0.26 | 0.20 |
| % of Variance Explained | 23.04% | 16.27% |
| Cronbach alpha | 0.64 | 0.40 |

Supplemental Table 4 Recovery Time Mixed Ordinal Regression Models

| **Variables** | **Recovery Time OR (95% CI)** | | | |  |  |
| --- | --- | --- | --- | --- | --- | --- |
|  | **Model 1** | **Model 2** | **Model 3** | **Model 4** | **Model 5** | **Model 6** |
| Age | 1.00 (0.96, 1.04) | 1.00 (0.96, 1.04) | 1.01 (0.97, 1.04) | 1.00 (0.96, 1.04) | 1.01 (0.97, 1.04) | 1.01 (0.97, 1.05) |
| Sex  (Female vs Male) | 1.12 (0.40, 3.14) | 1.05 (0.36, 3.01) | 0.87 (0.32, 2.33) | 0.93 (0.33, 2.67) | 0.85 (0.32, 2.27) | 0.83 (0.31, 2.24) |
| Center  (Calgary vs Hamilton) | 0.53 (0.19, 1.46) | 0.52 (0.19, 1.47) | 0.55 (0.21, 1.44) | 0.58 (0.21, 1.61) | 0.59 (0.23, 1.54) | 0.58 (0.22, 1.54) |
| Vintage (years) | 1.02 (0.92, 1.12) | 1.03 (0.93, 1.14) | 1.02 (0.93, 1.12) | 1.04 (0.94, 1.15) | 1.02 (0.93, 1.12) | 1.03 (0.93, 1.13) |
| CAD or PVD | 1.53 (0.53, 4.46) | 1.47 (0.49, 4.35) | 1.79 (0.65, 4.96) | 1.59 (0.54, 4.70) | 1.92 (0.70, 5.29) | 1.85 (0.66, 5.17) |
| Stroke | **0.21 (0.05, 0.99)** | 0.22 (0.05, 1.05) | 0.27 (0.06, 1.15) | 0.23(0.05, 1.07) | 0.26 (0.06, 1.12) | 0.27 (0.06, 1.16) |
| Diabetes | 1.37 (0.46, 4.06) | 1.25 (0.41, 3.77) | 1.12 (0.40, 3.14) | 1.27 (0.42, 3.79) | 1.22 (0.44, 3.40) | 1.15 (0.41, 3.24) |
| IDH | --- | **3.01 (1.18, 7.67)** | 2.31 (0.91, 5.85) | 2.47 (0.96, 6.34) | --- | 2.13 (0.84, 5.43) |
| Symptom Cluster Component 1 | --- | --- | **1.72 (1.32, 2.24)** | --- | **1.64 (1.25, 2.15)** | **1.62 (1.23, 2.12)** |
| Symptom Cluster Component 2 | --- | --- | --- | **1.38 (1.09, 1.77)** | 1.26 (0.99, 1.61) | 1.24 (0.97, 1.58) |
| AIC | 711.54 | 708.03 | 693.58 | 702.66 | 693.17 | 692.60 |

OR= Odds Ratio from mixed-effects ordinal regression models; CI= Confidence Interval; CAD= Coronary Artery Disease; IDH= intra-dialytic hypotension; PVD= Peripheral Vascular Disease; AIC = Akaike Information Criteria, sys=systolic

Supplemental Table 5 PCS Mixed Linear Regression Models

|  | **PCS β (95% CI)** | | | |
| --- | --- | --- | --- | --- |
| **Variables** | **Model 1** | **Model 2** | **Model 3** | **Model 4** |
| Age (years) | -0.00 (-0.09, 0.09) | -0.00 (-0.09, 0.10) | -0.00 (-0.09, 0.09) | -0.00 (-0.09, 0.09) |
| Sex  (Female vs Male) | **-3.38 (-5.87, -0.90)** | **-3.39 (-5.87, -0.90)** | **-3.10 (-5.49, -0.72)** | **-3.11 (-5.49, -0.72**) |
| Center  (Calgary vs Hamilton) | -1.16 (-3.57, 1.25) | -1.16 (-3.57, 1.25) | -1.18 (-3.47, 1.11) | -1.17 (-3.47, 1.12) |
| Vintage (years) | 0.08 (-0.16, 0.31) | 0.08 (-0.16, 0.31) | 0.10 (-0.13, 0.33) | 0.10 (-0.13, 0.33) |
| CAD or PVD | -1.13 (-3.70, 1.44) | -1.14 (-3.71, 1.44) | -1.27 (-3.73, 1.18) | -1.28 (-3.74, 1.18) |
| Stroke | 0.96 (-2.68, 4.59) | 0.97 (-2.68, 4.61) | 0.61 (-2.88, 4.09) | 0.62 (-2.87, 4.10) |
| Diabetes | -0.78 (-3.38, 1.82) | -0.78 (-3.38, 1.82) | -0.58 (-3.07, 1.90) | -0.58 (-3.07 1.90) |
| IDH | --- | 0.26 (-2.27, 2.79) | --- | 0.26 (-2.27, 2.79) |
| Symptom Cluster Component 1 | --- | --- | **-0.72 (-1.29, -0.15)** | **-0.72 (-1.29, -0.15)** |
| Symptom Cluster Component 2 | --- | --- | 0.19 (-0.45, 0.83) | 0.19 (-0.46, 0.83) |
| AIC | 1808.39 | 1810.35 | 1806.83 | 1808.79 |

PCS= Physical Component Score; β= Beta Coefficient from mixed-effects linear regression models; CI=Confidence Interval; CAD= Coronary Artery Disease; IDH= intra-dialytic hypotension; PVD= Peripheral Vascular Disease; AIC = Akaike Information Criterion, sys=systolic

Supplemental Table 6 MCS Mixed Linear Regression Models

| **Variables** | **MCS β (95% CI)** | | | |
| --- | --- | --- | --- | --- |
|  | **Model 1** | **Model 2** | **Model 3** | **Model 4** |
| Age | 0.07 (-0.03, 0.16) | 0.07 (-0.03, 0.17) | 0.05 (-0.04, 0.15) | 0.06 (-0.04, 0.15) |
| Sex  (Female vs Male) | -1.25 (-3.92, 1.43) | -1.26 (-3.95, 1.43) | -0.56 (-3.11, 1.98) | -0.58 (-3.13, 1.98) |
| Center  (Calgary vs Hamilton) | 0.76 (-1.83, 3.34) | 0.78 (-1.82, 3.38) | 0.79 (-1.64, 3.22) | 0.81 (-1.63, 3.25) |
| Vintage (years) | **-0.26 (-0.51, -0.00)** | **-0.26 (-0.51, -0.00)** | **-0.25 (-0.49, -0.01)** | **-0.25 (-0.50, -0.01)** |
| CAD or PVD | -0.37 (-3.14, 2.41) | -0.41 (-3.20, 2.38) | -0.41 (-3.02, 2.21) | -0.47 (-3.10, 2.17) |
| Stroke | -0.39 (-4.31, 3.53) | -0.34 (-4.28, 3.60) | -1.06 (-4.76, 2.64) | -1.00 (-4.72, 2.72) |
| Diabetes | 0.35 (-2.45, 3.15) | 0.35 (-2.46, 3.16) | 0.40 (-2.24, 3.04) | 0.41 (-2.25, 3.06) |
| IDH | --- | 1.00 (-2.10, 4.10) | --- | 1.19 (-1.85, 4.23) |
| Symptom Cluster Component 1 | --- | --- | **-0.81 (-1.47, -0.15)** | **-0.82 (-1.48, -0.16)** |
| Symptom Cluster Component 2 | --- | --- | -0.72 (-1.50, 0.07) | -0.72 (-1.50, 0.06) |
| AIC | 1908.49 | 1910.01 | 1899.43 | 1900.86 |

PCS= Physical Component Score; β= Beta Coefficient from mixed-effects linear regression models; CI=Confidence Interval; CAD= Coronary Artery Disease; IDH= intra-dialytic hypotension; PVD= Peripheral Vascular Disease; AIC = Akaike Information Criterion, sys=systolic

Supplemental Table 7 Relationship between Intra-dialytic Symptom Clusters, Intra-dialytic Hypotension and Recovery Time

|  | **Mixed-effects Linear Regression** | **Mixed-effects ordinal logistic regression** | | |
| --- | --- | --- | --- | --- |
| **Variables** | **Component 2**  **β (95% CI)** | **Recovery Time**  **OR (95% CI)** | **Recovery Time**  **OR (95% CI)** | **Recovery Time**  **OR (95% CI)** |
| Age | -0.01 (-0.03, 0.01) | 1.00 (0.96, 1.04) | 1.00 (0.96, 1.04) | 1.00 (0.96, 1.04) |
| Sex  (Female vs Male) | 0.42 (-0.00, 0.83) | 1.05 (0.36, 3.01) | 0.97 (0.35, 2.72) | 0.93 (0.33, 2.67) |
| Center  (Calgary vs Hamilton) | -0.28 (-0.69, 0.13) | 0.52 (0.19, 1.47) | 0.59 (0.21, 1.61) | 0.58 (0.21, 1.61) |
| Vintage (years) | **-0.05 (-0.09, -0.01)** | 1.03 (0.93, 1.14) | 1.03 (0.94, 1.14) | 1.04 (0.94, 1.15) |
| CAD or PVD | -0.18 (-0.62, 0.25) | 1.47 (0.49, 4.35) | 1.67 (0.58, 4.84) | 1.59 (0.54, 4.70) |
| Stroke | -0.12 (-0.73, 0.49) | 0.22 (0.05, 1.05) | 0.22 (0.05, 1.03) | 0.23 (0.05, 1.07) |
| Diabetes | -0.03 (-0.48, 0.41) | 1.25 (0.41, 3.77) | 1.37 (0.47, 4.02) | 1.27 (0.42, 3.79) |
| IDH | **0.53 (0.04, 1.03)** | **3.01 (1.18, 7.67)** | --- | 2.47 (0.96, 6.34) |
| Symptom Cluster Component 2 | --- | --- | **1.43 (1.12, 1.83)** | **1.38 (1.09, 1.77)** |
| AIC | 1006.77 | 708.03 | 704.32 | 702.66 |

β= Beta Coefficient from mixed-effects linear regression model; CI= Confidence Interval; OR = Odds Ratio from mixed-effects ordinal regression; CAD= Coronary Artery Disease; IDH= intra-dialytic hypotension; PVD= Peripheral Vascular Disease; AIC = Akaike Information Criteria

Supplemental Table 8 Symptom Cluster Component Scores and Intra-dialytic Hypotension from Mixed-effects Linear Regression Analysis

|  | **Symptom Cluster Score β (95% CI)** | |
| --- | --- | --- |
|  | **Cluster 1** | **Cluster 2** |
| IDH (Present vs Absent**)** | 0.32 (-0.08, 0.71) | **0.61 (0.11, 1.10)** |

β = Beta Coefficient; CI= Confidence Interval; IDH= intra-dialytic hypotension. Mixed-effects linear regression analysis run in records included in recovery time models

| **Variables** |  | **Recovery Time OR (95% CI)** | | | | | | | | | |
| --- | --- | --- | --- | --- | --- | --- | --- | --- | --- | --- | --- |
|  | **Model 1** | **Model 2** | **Model 3** | **Model 4** | **Model 5** | **Model 6** | **Model 7*** | **Model 8** | **Model 9** | **Model 10** | **Model 11** |
| Age | 1.00 (0.96, 1.04) | 1.00 (0.96, 1.04) | 1.00 (0.96, 1.04) | 1.00 (0.96, 1.04) | 1.00 (0.96, 1.04) | 1.00 (0.96, 1.04) | 1.00 (0.97, 1.04) | 1.00 (0.96, 1.04) | 1.00 (0.96, 1.04) | 1.00 (0.96, 1.05) | 1.00 (0.96, 1.04) |
| Sex  (Female vs Male) | 1.05 (0.36, 3.01) | 1.00 (0.35, 2.82) | 0.99 (0.34, 2.88) | 1.03 (0.36, 2.92) | 1.05 (0.37, 3.03) | 0.96 (0.34, 2.71) | 1.05 (0.40, 2.80) | 1.00 (0.35, 2.85) | 0.98 (0.35, 2.71) | 0.82 (0.28, 2.37) | 1.00 (0.35, 2.82) |
| Center  (Calgary vs Hamilton) | 0.52 (0.19, 1.47) | 0.50 (0.18, 1.38) | 0.55 (0.19, 1.55) | 0.55 (0.20, 1.53) | 0.53 (0.19, 1.49) | 0.58 (0.21, 1.60) | 0.54 (0.21, 1.41) | 0.55 (0.20, 1.54) | 0.51 (0.19, 1.38) | 0.52 (0.18, 1.44) | 0.52 (0.19, 1.44) |
| Vintage (years) | 1.03 (0.93, 1.14) | 1.01 (0.91, 1.12) | 1.03 (0.93, 1.14) | 1.03 (0.93, 1.14) | 1.03 (0.93, 1.14) | 1.03 (0.93, 1.14) | 1.03 (0.94, 1.13) | 1.03 (0.93, 1.14) | 1.02 (0.92, 1.12) | 1.03 (0.93, 1.14) | 1.03 (0.93, 1.14) |
| CAD or PVD | 1.47 (0.49, 4.35) | 1.48 (0.51, 4.31) | 1.43 (0.48, 4.28) | 1.49 (0.51, 4.38) | 1.53 (0.51, 4.56) | 1.58 (0.54, 4.63) | 1.47 (0.54, 4.03) | 1.51 (0.52, 4.45) | 1.58 (0.55, 4.53) | 1.70 (0.57, 5.04) | 1.58 (0.54, 4.63) |
| Stroke | 0.22 (0.05, 1.05) | 0.21 (0.05, 0.98) | 0.21 (0.04, 1.03) | 0.23 (0.05, 1.08) | 0.22 (0.05, 1.05) | 0.23 (0.05, 1.06) | 0.26 (0.06, 1.12) | 0.22 (0.05, 1.05) | 0.25 (0.05, 1.13) | 0.20 (0.04, 0.96) | 0.26 (0.06, 1.24) |
| Diabetes | 1.25 (0.41, 3.77) | 1.17 (0.39, 3.46) | 1.27 (0.42, 3.87) | 1.23 (0.41, 3.68) | 1.30 (0.43, 3.93) | 1.24 (0.42, 3.67) | 1.31 (0.47, 3.66) | 1.22 (0.41, 3.64) | 1.14 (0.39, 3.31) | 1.16 (0.38, 3.48) | 1.16 (0.39, 3.45) |
| IDH | **3.01 (1.18, 7.67)** | **3.14 (1.23, 8.03)** | **3.01 (1.18, 7.68)** | **2.81 (1.10, 7.16)** | **2.91 (1.14, 7.46)** | **2.70 (1.05, 6.91)** | 2.35 (0.94, 5.86) | **2.74 (1.06, 7.07)** | **3.02 (1.19, 7.70)** | 2.42 (0.94, 6.20) | **2.71 (1.06, 6.94)** |
| Bone or Joint Pain | --- | **1.50 (1.05, 2.14** | --- | --- | --- | --- | --- | --- | --- | --- | --- |
| Chest Pain | --- | --- | 2.73 (0.80, 9.30) | --- | --- | --- | --- | --- | --- | --- | --- |
| Diarrhea | --- | --- | --- | 1.71 (0.87, 3.35) | --- | --- | --- | --- | --- | --- | --- |
| Headache | --- | --- | --- | --- | 1.19 (0.75, 1.89) | --- | --- | --- | --- | --- | --- |
| Feeling Nervous | --- | --- | --- | --- |  | 1.74 (0.88, 3.41) | --- |  |  |  |  |
| Lack of Energy | --- | --- | --- | --- | --- | --- | **1.56 (1.20, 2.02)** | --- | --- | --- | --- |
| Muscle Cramps | --- | --- | --- | --- | --- | --- | --- | 1.20 (0.85, 1.69) | --- | --- | --- |
| Muscle Soreness | --- | --- | --- | --- | --- | --- | --- | --- | **1.62 (1.14, 2.29)** | --- | --- |
| Nausea/Vomiting | --- | --- | --- | --- | --- | --- | --- | --- | --- | **2.25 (1.33, 3.81)** | --- |
| Shortness of Breath | --- | --- | --- | --- | --- | --- | --- | --- | --- | --- | **1.64 (1.05, 2.54)** |
| AIC | 708.03 | 704.80 | 707.48 | 707.49 | 709.49 | 707.47 | 699.09 | 708.96 | 702.44 | 700.03 | 705.06 |

Supplemental Table 9 Recovery Time Models with Individual Symptoms

PCS= Physical Component Score; OR= Odds Ratio from mixed-effects ordinal models; CI=Confidence Interval; CAD= Coronary Artery Disease; IDH= intra-dialytic hypotension; PVD= Peripheral Vascular Disease; AIC = Akaike Information Criterion, sys=systolic


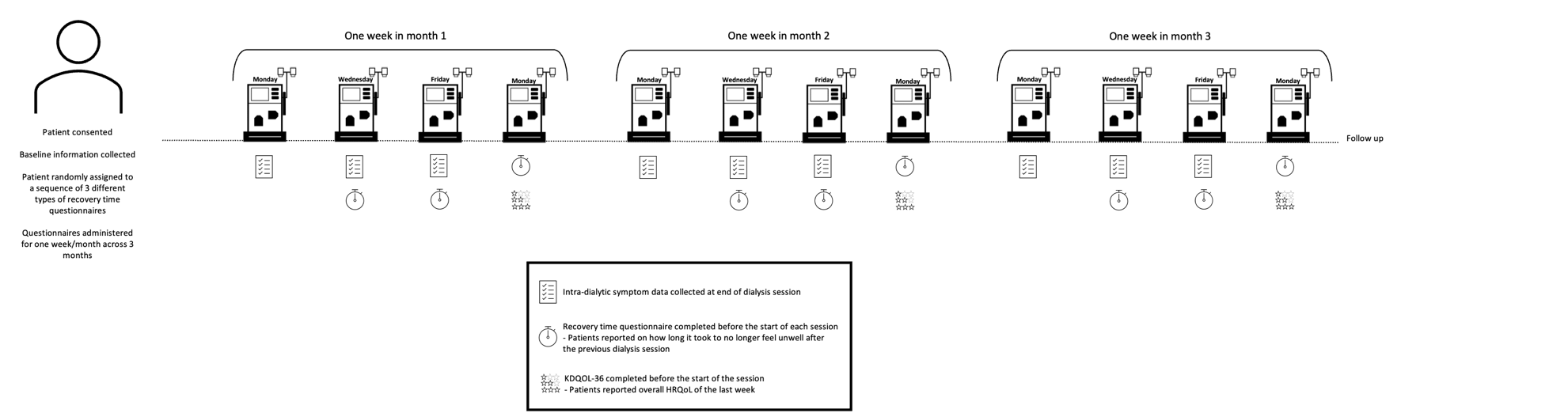


Supplemental Figure 1: Overview of Original Study Schedule. Example study schedule for patient receiving dialysis on a weekly Monday, Wednesday and Friday schedule.

Supplemental Figure 2: Principal Component Analysis Scree Plot
